# A community engaged primary healthcare strategy to address rural school student inequities: a descriptive paper

**DOI:** 10.1017/S1463423618000907

**Published:** 2019-03-20

**Authors:** Debra Jones, Jacqueline Ballard, Robert Dyson, Peter Macbeth, David Lyle, Palatty Sunny, Anu Thomas, Indira Sharma

**Affiliations:** 1 The University of Sydney Director Primary Health Care, Broken Hill University Department of Rural Health, Broken Hill, Australia; 2 Nursing and Midwifery Directorate, Nurse Manager Policy Practice and Initiatives, Far West Local Health District, Broken Hill, Australia; 3 Networked Specialist Centre Facilitator, New South Wales Department of Education, Broken Hill, Australia; 4 Far West Network, NSW Department of Education Director Educational Leadership, Broken Hill, Australia; 5 The University of Sydney Head of Department, Broken Hill University Department of Rural Health, Broken Hill, Australia; 6 Far West Local Health District Primary Health Care Registered Nurse: Schools-Based, Bachelor of Nursing, Broken Hill, Australia

**Keywords:** Australia, children and adolescents, community engagement, health and well-being, primary healthcare, rural

## Abstract

**Aim:**

This descriptive paper aims to describe the design and implementation of a community engaged primary healthcare strategy in rural Australia, the Primary Healthcare Registered Nurse: Schools-Based strategy. This strategy seeks to address the health, education and social inequities confronting children and adolescents through community engaged service provision and nursing practice.

**Background:**

There have been increasing calls for primary healthcare approaches to address rural health inequities, including contextualised healthcare, enhanced healthcare access, community engagement in needs and solutions identification and local-level collaborations. However, rural healthcare can be poorly aligned to community contexts and needs and be firmly entrenched in health systems, marginalising community participation.

**Methods:**

This strategy has been designed to enhance nursing service and practice responsiveness to the rural context, primary healthcare principles, and community experiences and expectations of healthcare. The strategy is underpinned by a cross-sector collaboration between a local health district, school education and a university department of rural health. A research framework is being developed to explore strategy impacts for service recipients, cross-sector systems, and the establishment and maintenance of a primary healthcare nursing workforce.

**Findings:**

Although in the early stages of implementation, key learnings have been acquired and strategic, relationship, resource and workforce gains achieved.

## Introduction

The conditions in which children and adolescents grow, live and learn impact their health and well-being (Commission on the Social Determinants of Health, 2008). Geographical isolation, lower socio-economic status, disengagement from school education and poorer health status have adverse impacts on the health, educational and social outcomes for rural Australian children and adolescents (Bankwest Curtin Economics Centre, 2017). Whilst significant Australian health investments are targeted towards the first five years of life and adulthood, fewer investments are directed towards the provision of comprehensive healthcare for school-aged students, specifically those residing in rural locations (Viner *et al*., 2012).

In addressing these challenges there have been increasing calls for primary healthcare approaches that; promote contextualised healthcare, enhance healthcare access, engage communities in the identification of their healthcare needs and solutions, and support local-level cross-sectorial collaborations. However, healthcare can lack adaptability to local contexts and be poorly aligned to community needs. Furthermore, healthcare decision making can be centralised and controlled within health systems, marginalising community participation in their own healthcare agendas. There is a requirement for new approaches to how we design, implement and evaluate rural primary healthcare innovations if we are to make substantive and sustainable health improvements for rural populations (Alderwick *et al*., 2016).

This descriptive paper aims to describe the design and implementation of a community engaged primary healthcare strategy in rural New South Wales, Australia, the Primary Healthcare Registered Nurse: Schools-Based (PHCRN:SB) strategy. This strategy seeks to address the health, education, and social inequities confronting rural children and adolescents through community engaged primary healthcare service design and implementation. This strategy aligns nursing services and practice to the unique rural context, the principles of primary healthcare [World Health Organization (WHO), 2008] and community experiences and expectations of healthcare for children, adolescents and their families. The strategy is underpinned by a cross-sector collaboration between a local health district, school education and a university department of rural health. Although in the early stages of implementation, key learnings have been acquired and strategic, relationship, resource and workforce gains achieved.

### The PHCRN:SB strategy

The PHCRN:SB strategy, whilst sharing some similarities with contemporary school nursing services (Maughan *et al*., 2016), differs significantly in why and how the strategy was designed, who was engaged in this design, and the processes associated with strategy implementation. The PHCRN:SB strategy was intentionally designed to respond to the unique rural community context, was informed by community experiences and expectations of healthcare for children, adolescents and their families, and seeks to align nursing practice and service provision to the principles of primary healthcare.

Five new full-time primary healthcare registered nursing positions have been established to deliver health promotion programs, enhance healthcare access through the earlier identification of needs and service activation, and contribute to service coordination and integration for children and adolescents already experiencing complex/chronic conditions. These positions are co-located on primary school campuses and transition into secondary school settings with the intent to establish and maintain healthcare and health professional relationship consistency and continuity (Far West Local Health District, 2017).

Traditional approaches to the design of healthcare strategies can promote decontextualized care, be linear in approach and centralised and controlled by health systems (Hawe, 2015). In contrast, this strategy is responsive to context, leveraging off existing schools-based health programs and established relationships, and informed by findings from extensive community consultations. Key stakeholders engaged in the design and implementation of this strategy have also been highly sensitive to the need to ensure the strategy: was underpinned by theory and evidence; drew on the skills and scope of practice of the right health professional to deliver on strategy aims and community expectations; responsive to the needs of individual school communities; receptive to adaptation as the strategy was being implemented and new learnings acquired; evaluated in terms of strategy effectiveness for a range of stakeholders; and appropriately governed.

### Contextualising the strategy

Primary healthcare strategies require an understanding of the context in which the strategy is being introduced if substantive health improvements are to be achieved (Campbell *et al*., 2000). The conditions that contribute to poor health and wellbeing are multifaceted and in such circumstances ‘it seems unrealistic to expect solutions to emerge from any single agency, organisation, or social sector’ (Henig *et al*., 2016: 4). However, health strategies can ‘be conceptualized as having some reality of their own, without particular context’ (Paterson *et al*., 2009: 3).

This strategy builds upon an established cross-sector collaboration between school education, a local health district, and a university department of rural health (Jones *et al.*, 2015). These partners have collaborated since 2008 in the design, implementation and evaluation of service-learning programs that align allied health student clinical placements to student-led service provision for children experiencing development vulnerabilities and inequity of access to services, addressing a community identified need (Jones *et al*., 2016). The longevity of the collaboration has enabled the consolidation of trusting relationships, contributed to the redistribution of resources and community receptiveness to engaged healthcare and alternative healthcare solutions (Authors suppressed for review). These processes have highlighted the importance of ‘consulting local people about what matters to them’ (Alderwick *et al*., 2016: 31).

### Community engagement: community concerns, experiences and expectations of healthcare for children, adolescents and families

Primary healthcare includes health promotion, disease prevention, early identification of needs and healthcare responses (WHO, 2008). Primary healthcare practice acknowledges the importance of community engagement to increase our understanding of health needs and to improve health outcomes. The Centers for Disease Control and Prevention define community engagement as ‘the process of working collaboratively with and through groups of people affiliated by geographical proximity, special interest, or similar situations to address issues affecting the well-being of those people’ (2011: 7). Community engaged healthcare is considered necessary to ensure: community voices inform their healthcare agendas; realistic and appropriate healthcare services and; service alignment to community need and expectations of healthcare (Lin *et al*., 2014). Whilst community engaged primary healthcare is considered desirable in rural contexts (Hyett *et al*. 2014), significant challenges exist in achieving this level of engagement (Kernick, 2004; WHO, 2008).

In extending on the work of this collaboration, extensive community consultations were undertaken to identify additional healthcare concerns for children and adolescents. Additional community identified concerns included limited community engagement in the identification of health needs and solutions, fragmented service provision and the provision of services at a distance from where needs exist, schools. The increasing complexity confronting families in navigating health services and a lack of focus on health promotion, early identification of needs and service activation were additional concerns.

Community perspectives of unmet health needs included the poor mental health and wellbeing of children and adolescents, limited support of families with school students already experiencing complex and chronic conditions and social concerns associated with student exposure to traumatic life events. Previous community experiences of healthcare included illness orientated approaches and service provision that addressed service needs in preference to community needs.

Community expectations of healthcare included care that was focused on health promotion, the early identification of needs and service activation. Additional expectations included integrated and coordinated care centred on the needs of service recipients and families, including service responsiveness to families that have or continue to experience trauma in their lives.

These findings informed the following responses by cross-sector partners: (1) an exploration of evidence and theory; (2) the identification of the right health professional with the right scope of practice to provide services to address these findings; and (3) the design and implementation of a potential solution, the PHCRN:SB strategy.

### Exploring the evidence and theories

Guided by these findings, an exploration of the literature focused on the principles associated with primary healthcare and integrated care and the theories of family-centred care (FCC) and trauma-informed care. Primary healthcare principles include community participation in promoting health and addressing health inequities and cross-sectorial collaborations. Within primary healthcare, resources and actions are directed towards populations that experience the greatest levels of inequities (WHO, 2008), in this instance rural children and adolescents. Integrated care principles include the provision of care that is co-ordinated and integrated across different providers and settings, care that tackles the social determinants of ill-health through cross-sector collaborations and care that is co-produced through partnerships with communities (Minkman, 2016).

FCC locates ‘the needs of the child, in the context of their family and community’ through the development of collaborative and dynamic models of care (MacKean *et al*., 2005: 74). FCC is considered to be accessible, coordinated, comprehensive, culturally competent, continuous and compassionate (Hagan *et al*., 2008). Benefits of FCC include the establishment of trusting and respectful relationships between health providers and families, enhanced communication and understanding of family needs and the provision of care that is aligned to the needs of children and families (Kuo *et al*., 2012).

The Australian Childhood Foundation stated that trauma is ‘the emotional, psychological and physiological residue left over from heightened stress that accompanies experiences of threat, violence, and life-challenging events’ (2010: 10). Wall *et al*. stated that a ‘program, organisation or system that is trauma-informed realises the widespread impact of trauma and understands potential pathways for recovery’ (2016: 6). Trauma-informed practices aim to support children and adolescents to reset their internal stress and arousal levels.

In responding to community expectations of providing healthcare closer to where needs exist, literature and evidence associated with the provision of healthcare services on school campuses was also explored. Whilst significant evidence of school-based healthcare strategies existed at the international level (Leroy *et al*., 2017), less evidence was available that described the role of communities in informing the design, implementation and adaptation of these services. A lack of Australian evidence, specifically evidence informed by rural communities was also identified. Schools-based healthcare literature also reflected a discrete approach to service provision that targeted either primary or secondary school aged students with services being delivered by different health professionals working independently to each other (Leroy *et al*., 2017). This approach to service provision was perceived by local partners to have the potential to contribute to fragmented healthcare and a lack of service and relationship continuity for families. This evidence, principles and theories have informed the design of a conceptual model of nursing care to guide the practice of the PHCRNs within this strategy. This model of care will be described in detail in future publications.

### Identifying the right health professional with the right scope of practice

In designing a healthcare strategy capable of responding to the rural context, strategy aims and community expectations, there was an imperative to identify the right health professional with the right scope of practice to address the clinical and non-clinical concerns identified. Pragmatically, a number of professions were excluded from consideration based on; existing levels of schools-based allied health service provision, diversity of needs identified and the need to ensure sustained funding for the additional health professionals.

The exploration of health professional scopes of practice and the school-based healthcare literature identified synergies between the strategy, community expectations and the scope of practice of PHCRNs (Carryer *et al*., 2015). Carryer *et al*. stated that ‘the increasing importance of conceptualizing health and wellbeing…necessitates a holistic perspective and signals the importance of nurses in primary health care’ (2015: 151). PHCRNs can provide health promotion, education and illness prevention services as well as clinical support for individuals and families experiencing complex and chronic conditions. PHCRNs act as linking mechanism between services to improve population health and care integration (Keleher *et al*., 2010; Australian College of Nursing, 2015). The connectivity of PHCRNs, specifically those located on school campuses, provides a unique perspective on how best to address the health and education needs of children and adolescents (Concepcion *et al*., 2007).

### Strategy responsiveness to schools

Whilst local schools may share some similarities, they each capture a unique set of epidemiological, socio-cultural, ethical, political and socio-economic conditions. Therefore, healthcare approaches that may work in one school may not apply to another. In ensuring strategy responsiveness, partners reached a consensus that services would need to be flexible enough to account for whole of school community health strategies as well as tailored strategies that could better meet the needs of individual schools and students (Kreuter and Skinner, 2000). The combination of population targeted and tailored strategies ‘can be more effective than generic interventions which do not take into consideration the characteristics of those to whom they will be offered’ (Kreuter and Skinner, 2000: 1).

### Strategy responsiveness to adaptation

A developmental evaluation approach has been adopted in the early stages of strategy design and implementation. Developmental evaluation supports the design and implementation of healthcare innovations in complex environments and is responsive to non-linear dynamics. Social innovations, such as the PHCRN:SB strategy, can be considered non-linear pathways to change, experiencing dynamic interactions, unexpected and unanticipated divergences. ‘Developmental evaluation adapts to the realities of nonlinear dynamics rather than trying to impose order and certainty on a disorderly and uncertain world’ (Patton, 2011: 5).

### Strategy evaluation and research

Three key research streams associated with the strategy have been identified for exploration: (1) service recipient impacts and outcomes; (2) cross-sector systems impacts and; (3) implications for the establishment and maintenance of a rural Australian primary healthcare nursing workforce. Each of these streams has a number of key focus areas. Stream 1 will explore education, health and social impacts for service recipients (students, families and schools). Stream 2 will explore strategic, policy, funding and practice impacts on cross-sector systems. Stream 3 will explore impacts for the PHCRNs, the influence of the nursing model of care on nursing practice and pre-registration student experiences within the strategy. Each stream has cross-sector research leaders with responsibility to convene research sub-groups, develop research protocols and collectively contribute to the overarching research agenda.

### Strategy governance

Governance structures play a critical role in promoting the effectiveness and success of healthcare innovations. Governance involves ‘ensuring strategic policy frameworks exist and are combined with effective oversight, coalition building, regulation, attention to system design and accountability’ (WHO, 2007: 86). A cross-sector governing committee, inclusive of community and Indigenous representation, has been established to provide strategic oversight for this strategy. The roles of this governance committee include: the endorsement of collective aims and goals associated with the strategy; contribution to open and transparent communication and planning processes across key stakeholders and the broader community; identification of solutions to address healthcare gaps and barriers to healthcare access; and the provision of cultural guidance to ensure strategy responsiveness to the rural context and Indigenous populations.

## Key learnings acquired

A number of conceptual, cross-sector, health, professional, financial and research learning points have been gained. Conceptually, it became evident early in community consultations that the title ‘school nurse’ was not considered sufficient in describing the role and scope of nursing practice. Whilst synergies exist between contemporary school nursing practices, strategy aims, and the role of the PHCRNs, significant differences were identified (ie, a life-phases approach to school student health, the engagement of community in co-designing strategy and service, and the explicit focus on FCC, trauma-informed care, integrated care and primary healthcare provision). The title, PHCRN:SB, was adopted to: clarify the role and scope of nursing practice; respond to community perceptions and feedback; and provide a contemporary interpretation of nursing care and practice within a rural Australian and school-based settings.

Key cross-sector learning points included the requirement for significant time investments to establish a consensus-orientated (Johnston *et al*., 2011) interpretation of strategy intent. Given the mutually reinforcing nature of health and education on life outcomes, discussions were held on how best to define the strategy within and across these sectors. Established differences can contribute to how social challenges are framed and addressed (Selsky and Parker, 2010). To ‘find consensus and create social and economic value, leaders of a collaborative initiative must be able to relate the interests and actions of individual agents to wider, system level relationships’ (Fratantuono and Sarcone, 2017: 5).

Despite wide spread support for the strategy, concerns were raised in relation to the potential for the PHCRNs to contribute to service fragmentation, increased referrals and workloads for other services. In contrast, the literature consistently identified the role of PHCRNs in promoting care coordination and integration. Cross-sector stakeholders acknowledge that tensions can be created when new health strategies have the potential to disrupt existing status quos and healthcare arrangements (North West Joint Improvement Partnership, 2010).

Professionally, challenges have been experienced in attracting suitably qualified registered nurses to the positions. Additional investments have been required to support the appointed PHCRNs in transitioning from hospital-centric and acute care practice to schools-based and primary healthcare practice. Substantial resource, time and education investments have been directed towards nurse preparation for primary healthcare practice, including the enrolment of all nurses in post-graduate primary healthcare coursework.

The uniqueness of the opportunity to co-design and contextualise nursing care and practice has been described by nursing executives and academics. Financially, the need to secure sustainable funding to appoint the PHCRNs has been a focus of the collaboration. This focus has been informed by previous experiences of short-term funding, lack of sustainability of new initiatives in rural contexts and levels of community cynicism towards poorly resourced healthcare innovations.

## Gains to date

Although in the early stages of implementation, strategic, relationship, resource and workforce gains have already been achieved. Strategically, a cross-sector governance committee has been established and convened to provide guidance and executive level endorsement of strategy aims, nursing care and practice approaches. Strategic documents have been developed and endorsed by this committee, including strategy proposal, communication strategy, comprehensive literature reviews, an initial evaluation and research framework, and a new model of nursing care. Relationships across sectors at the executive, senior management and service levels have been established or consolidated with relationship consistency being promoted through structured and routine multi-level committee, management and service meetings. New and sustained resources to fund the five PHCRN positions have been secured through the local health district, contributing to service continuity, consistency and sustainability. All five nursing positions have now been appointed to.

## Conclusion

Evidence indicates that to effectively address the challenges confronting disadvantaged children and adolescents we need to build supportive services that coordinate care across agencies in the same community, specifically those engaging with the same families. Increasing our understanding of the health needs of children and adolescents, and the application of this knowledge in the development of responsive primary healthcare policies and practices is necessary in establishing safe, health promoting environments and improved health outcomes (Viner *et al*., 2012; Moore *et al*., 2015).

As the complexity of health, educational and social needs of rural school students increases, there are calls for greater levels of innovation in the design and implementation of alternative healthcare models that enhance service accessibility, acceptability and sustainability. Social innovations, those that have the capacity to transform the lives of individuals and communities, are required to resolve complex health inequities (Phills *et al*., 2008). Healthcare innovations, those led by rural communities, are more likely to address the needs of greater numbers of community members than those that are externally developed and driven. The PHCRN:SB strategy significantly challenges illness orientated, hospital-centric, de-contextualised healthcare and nursing practice within a rural Australian context. This strategy has the potential to transform the lives of some of Australia’s most marginalised children, adolescents and families and inform future approaches to rural healthcare design and the development of a primary healthcare ready nursing workforce.
